# Cutis marmorata telangiectatica congenita in a preterm newborn – Case report and literature review

**Published:** 2012-09-30

**Authors:** A Matic, S Pricic, Milan Matic, G Velisavljev Filipovic, A Ristivojevic

**Affiliations:** 1 Neonatology department, Institute for child and youth health care of Vojvodina, Novi Sad, Serbia; 2 Clinical centre of Vojvodina, Dermatovenereological clinic, Novi Sad, Serbia; 3 Neonatology department, Gynaecologic-obstetric clinic, Clinical centre of Vojvodina, Novi sad, Serbia

**Keywords:** Cutis marmorata telangictatica congenital, Van Lohuizen syndrome

## Abstract

**Background:**

Cutis marmorata telangiectatica congenita (CMTC) is a sporadic congenital skin vascular abnormality. Significant number of patients has other congenital anomalies.

**Case Report:**

We report a case of a preterm male newborn with cutis marmorata pattern presented on the skin of the face, right side of front of the trunk, whole back, glutei and both legs. Besides, microretrognatia and asymmetric, bad-formed, low-set ears were notable. Ophthalmologic findings showed visual impairment and pale optic nerve papilla. Monitoring of child showed mental underdevelopment and motor retardation.

**Conclusion:**

CMTC is a rare finding with good prognosis of skin malformations, with an obligation of dermatologist and paediatrician to investigate other associated congenital anomalies.

## Introduction

Cutis marmorata telangiectatica congenita (CMTC) is a rare, sporadic vascular abnormality of the skin of unknown aetiology, usually present at birth. It is characterized by the presence of localized or generalized persistent skin changes of cutis marmorata type and telangiectasia. Those typical skin lesions may be accompanied by ulcerations or skin atrophy. Skin changes, in most cases, show a tendency of spontaneous resolution in later life. The disease was first described in 1922 by van Lohuizen.(1) About 300 cases were described in the literature so far.

The diagnosis of CMTC can be established on the basis of clinical symptoms alone. Histological analysis shows numerous dilated capillaries and venules in the dermis level, but it is usually not necessary to confirm the diagnosis by skin biopsy.(2,3)

An association of CMTC with other congenital anomalies was described in a significant number of patients. The frequency of occurrence of coexisting congenital anomalies varies from 20% to 80% in the literature. Asymmetry of the body (usually hyper-, or hypo-plasia of the extremities), other vascular anomalies, glaucoma, psychomotor or mental retardation were usually described.(3,5,6)

## Case Report

Prematurely born newborn male of 31+4 gestational weeks was admitted on neonatal intensive care unit shortly after birth, because of respiratory distress syndrome requiring surfactant therapy and mechanical ventilation. He was born as a forth child from a non-consanguineous marriage, after insufficiently-controlled pregnancy of a healthy 28-year old mother. After delivery by caesarean section performed because of mothers haemorrhage, he was born in birth asphyxia – Apgar-score was 2/4. His birth weight was 1420g (25 percentiles), birth length 40cm (25 percentiles), head circumference 28,5cm (25 percentiles) – all in eutrophic range for his gestational age. Because of many diseases, primarily associated with prematurity (RDS, intracranial haemorrhage grade III, neonatal jaundice) our patient was treated at the Institute for child and youth health care of Vojvodina.

Skin changes were observed at birth, in the form of hyperpigmented macules of reticular pattern as well as telangiectasia. These changes did not disappear during the warming of child. The lesions were located on the face (cheeks, nose and above the right eye), right half of the front of the trunk (with a clear demarcation from the unchanged skin right in the midline), the entire back, gluteus and both legs and both arms. At birth, described skin changes have covered a total of 75% of the entire skin surface.

In addition, one could observed microretrognatia, asymmetric, poorly formed, low-set ears, eyebrows synophris, easily bent 5th finger, the increased distance between the thumb and second finger on his right foot; total minor malformation score was 4.

Regularly monitored laboratory, haematological and biochemical analysis were in accordance with the above diagnoses for which the child was treated at the Institute. Repeated ultrasound examinations of the head, except intracranial haemorrhage grade III, which was associated with prematurity and neonatal asphyxia, showed no anatomical anomalies of the central nervous system. Abdomen ultrasound and echocardiogram findings were unremarkable. Ophthalmic findings, except of retinopathy of prematurity (ROP) of grade II, were also unremarkable. The laboratory testing of function of the thyroid gland was within physiological range. Child's karyotype was 46, XY - normal male.

**Fig. 1:**
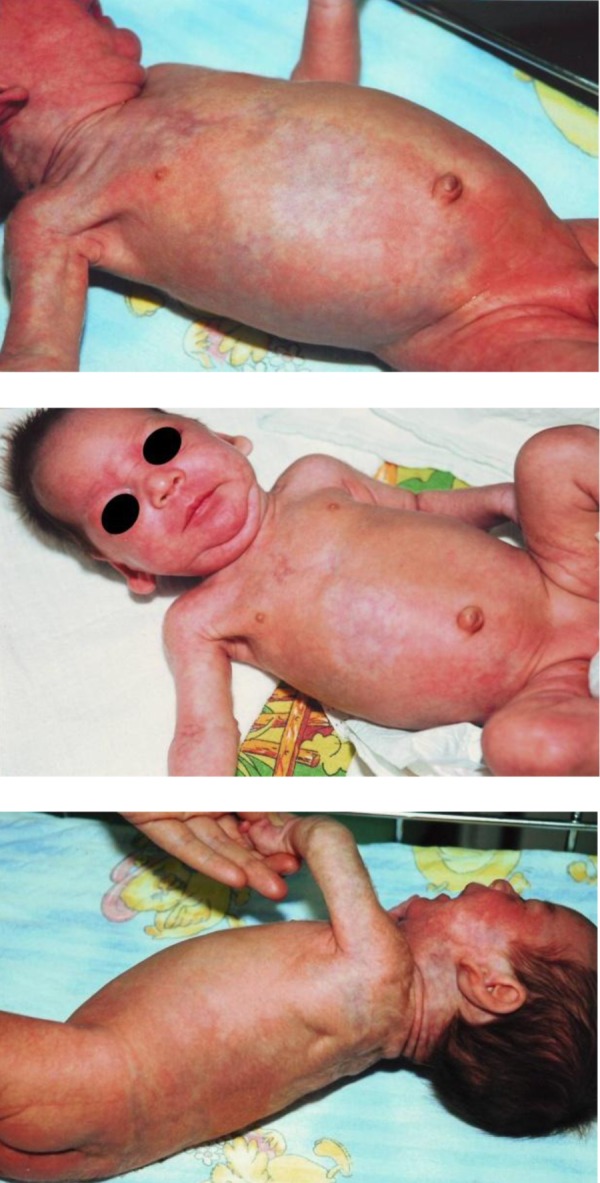
skin changes of a premature infant aged 14 days

After hospital discharge, parents were advised on regular follow-up of the child, according to Institutes protocol for prematurely born children, but they didn't come on any of the suggested exams. At the age of 10 months, local outpatient physician has sent the child at Institute because of lower respiratory tract infection; at the same time, distinct delay in psychomotor development was noticed.

At that point of time, exam of the child showed a partial but substantial regression of skin changes. At the age of 10 months, there were a total of 39% of the affected skin surface. The changes completely disappeared from both arms and left leg. Asymmetry of the body was not observed. The child's head was dyscranic and mycrocephalic, with head circumference of 39 cm – under 3 percentiles. The psychomotor development of the child was significantly impaired. At the age of 10 moths, psychological testing showed no social contact with child. Global development coefficient according to Brunet-Lezine scale was RQ = 35, impaired most severely in coordination (16) and social (22), less in motor (59) and language (55) area of development. Electroencephalography revealed focal epileptiform changes. He didn’t follow nor fix objects with his eyes; there were impression of severe vision impairment. Ophthalmologic exam showed pale optic nerve papilla, and visual evoked potentials were suggested, but parents refused the test. He also didn't respond to sound stimuli, and transiently evoked otoacoustic emissions showed hearing impairment.

**Fig. 2:**
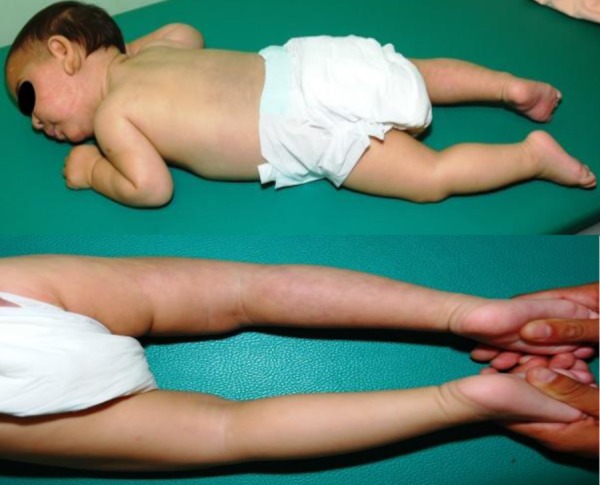
skin changes of a premature infant aged 10 months

## Discussion

Changes in CMTC are mostly present at birth, or they occur in the first few days after birth, as was the case in our patient. In a few larger series of cases of CMTC in the literature, appearing of skin changes at birth is reported in 93-94% of cases.(3-6) In these studies almost equal representation of both sexes was also observed.

According to distribution of skin changes, CTMC can be generalized or localized. It is not clear in what percentage localized forms occurs, but it is considered that they are more common, affecting about 60% children with CMTC, while generalized forms occur in about 40%.(5) The most common sites of involvement of localized forms of CMTC are limbs, especially legs, and torso. Complete, diffuse covering of entire skin has not been described in the available literature. In generalized form, besides trunk and limbs, face and scalp can be afflicted, while the palms, soles and mucous membranes are usually spared.(6-8) When skin changes are localized at the abdomen, a clear demarcation at the midline is practically always observed; this was the case with our patient. This case represents generalised form of CMTC, affecting rather large skin surface.

A variety of associated anomalies in CMTC are described in the literature. In a review of reported cases up to date, in which were included all studies with more than 10 cases, Kienast and Hoeger have found the presence of various associated anomalies in 132 out of 215 patients (61%),(4) with a variation from 18.8% (5) up to 80% among analysed studies (3). They have found that the asymmetry of the body is the most frequent (25.1%), then vascular anomalies (23.2%), skin atrophy (5.6%), neurological complications (5.1%), while ocular changes, skin ulcers and syndactyly were reported in a few cases.(4) In other larger series of cases, similar frequency of congenital malformations, by number as well as by type of anomaly, was observed. (9-11)

In 1997 Clayton-Smith et al.(12) and Moore et al.(13) simultaneously, but independently of one another, described a particular subgroup of CMTC, macrocephaly- CMTC (M-CMTC) syndrome. In those two studies a total of 22 children with macrocephaly, CMTC, naevus flammeus on the upper lip and philtrum and syndactyly were described. Among these patients, neurological disorders and other complications, of which some are potentially life threatening, were commonly observed. Besides, numerous other congenital malformations can occur as a part of this syndrome.(15) In the Macrocephaly- CMTC the most characteristic cutaneous vascular anomalies are the capillary malformations rather than Cutis Marmorata Telangiectatica Congenita as initially described. For that reason, it is now named as Macrocephaly-capillary malformation syndrome (M-CM). Currently, more than 130 cases of M-CM have been reported.(16)

Differential diagnosis of CMTC is rarely difficult because of distinctive appearance of skin changes. In the differential diagnosis one should think about the physiological cutis marmorata. It is a consequence of low temperatures and can be seen on the skin of healthy children, but is lost during the warm-up. Bockenheimerov syndrome (diffuse phlebectasia) should also always be taken in consideration. It is a rare progressive condition that is characterized by often large and painful venous ectasia, usually of a limb; this finding is not present at birth, but begins later in childhood. It is even more important to differentiate Klippel-Trenaunay syndrome (KTS), which is more common than CMTC. Patients with KTS have a vascular lesion, usually of naevus flammeus type with venectasia and hypertrophy of soft tissues; these findings are sometimes accompanied by internal hemangiomas. Although naevus flammeus is always present at birth, venectasia are not necessary visible during the first year of life. However, there is no spontaneous resolution of naevus flammeus during childhood, but rather it becomes more pigmented.(4,5) Disorders such as homocystinuria, Down syndrome, and de Lange syndrome are associated with cutis marmorata but other typical clinical features differs them from patients with CMTC.(3)

Diagnostic criteria for CMTC, which would have helped to clearly distinguish this disease from other vascular anomalies, have not yet been precisely defined. However, Kienast and Hoeger in 2009 suggested, on the basis of case series published in the literature and their own series, that three major (1: Congenital reticulate (marmorated) erythema; 2: Absence of venectasia; 3:Unresponsiveness to local warming) and two of the five minor criteria (1: Fading of erythema within 2 years; 2: Telangiectasia; 3:Port-wine stain outside the area affected by CMTC; 4: Ulceration; 5: Atrophy) is sufficient for the diagnosis of CMTC. Reticular erythema present at birth is a common denominator of all reported cases, so it has been considered major criteria. Unlike the physiological cutis marmorata, which occurs in otherwise healthy babies in a cold environment, CMTC does not respond to local warming. The absence of venectasia in the affected region of skin until the age of one year is very important finding, which differentiate CMTC from KTS. The presence of telangiectasia and ulcerations is optional and therefore can be classified as minor criteria; but if they are present, that speaks strongly in favour of CMTC. A gradual loss of erythema within 2 years occurs in more than 50% of patients; authors have also suggested that this phenomenon should be counted into the minor criteria.(4)

Our patient meets all three major and two minor criteria, and therefore we believe that it is undoubtedly a case of CMTC. When it comes to other findings – psychomotor delay, visual and hearing impairment, epileptiform findings on encephalography, there is no doubt that most of those are considerably connected with prematurity, neonatal asphyxia and high-grade intracranial hemorrhage. But, visual impairment as well as pale optic nerve papilla, which were observed at the age of 10 months, are not in accordance with finding of grade II ROP, especially because of evidence of improvement of ROP at the next ophthalmologic control performed before hospital discharge. Is there connection of these findings with CMTC is not clear. We couldn’t find such as coexisting problems with CMTC in literature data, but one cannot exclude such a possibility.

In conclusion CMTC is a rare finding with a good prognosis of skin changes, but it imposes an obligation on dermatologists and paediatricians to examine the presence of other potentially dangerous congenital anomalies. So, detailed and comprehensive examination of all the organs, as well as annual controls of skin changes and psychomotor development of these children is warranted.

## References

[1] Van Lohuizen CHJ (1922). Über eine seltene angeborene Hautanomalie (Cutis marmorata telangiectatica congenita).. Acta Derm Venereol.

[2] Fujita M, Darmstadt GL, Dinulos JG (2003). Cutis marmorata telangiectatica congenita with hemangiomatous histopathologic features.. J Am Acad Dermatol.

[3] Devillers ACA, de Waard-van der Spek FB, Oranje AP (1999). Cutis marmorata telangiectatica congenital: clinical features in 35 cases.. Arch Dermatol.

[4] Kienast AK, Hoeger PH (2009). Cutis marmorata telangiectatica congenita: a prospective study of 27 cases and review of the literature with proposal of diagnostic criteria.. Clin Exp Dermatol.

[5] Amitai DB, Fichmann S, Merlob P, Morad Y, Lapidoth M, Metzker A (2000). Cutis marmorata telangiectatica congenita: clinical findings in 85 patients.. Pediatr Dermatol.

[6] Picascia DD, Esterly NB (1989). Cutis marmorata telangiectatica congenita: report of 22 cases. J Am Acad Dermatol.

[7] Cohen PR, Zalar GL (1988). Cutis marmorata telangiectatica congenita:clinicopathologic characteristics and differential diagnosis.. Cutis.

[8] Rupprecht R, Hundeiker M (1997). Cutis marmorata telangiectatica congenita. Important aspects for dermatologic practice.. Der Hautarzt.

[9] Pehr K, Moroz B (1993). Cutis marmorata telangiectatica congenita: long-term follow-up, review of the literature, and reportof a case in conjunction with congenital hypothyroidism.. Pediatr Dermatol.

[10] Gerritsen MJ, Steijlen PM, Brunner HG, Rieu P (2000). Cutis marmorata telangiectatica congenita: report of 18 cases.. Br J Dermatol.

[11] Gelmetti C, Schianchi R, Ermacora E (1987). Cutis marmorata telangiectatica congenita. 4 new cases and review of the literature.. Ann Dermatol Venereol.

[12] Clayton-Smith J, Kerr B, Brunner H, Tranebjaerg L, Magee A, Hennekam RC, Mueller RF, Brueton L, Super M, Steen-Johnsen J, Donnai D (1997). Macrocephaly with cutis marmorata, haemangioma and syndactyly-A distinctive overgrowth syndrome.. Clin Dysmorphol.

[13] Moore CA, Toriello HV, Abuelo DN, Bull MJ, Curry CJ, Hall BD, Higgins JV, Stevens CA, Twersky S, Weksberg R, Dobyns WB (1997). Macrocephaly-cutis marmorata telangiectatica congenita: A distinct disorder with developmental delay and connective tissue abnormalities.. Am J Med Genet.

[14] Katugampola R, Moss C, Mills C (2008). Macrocephaly-cutis marmorata telangiectatica congenita: a case report and review of salient features.. J Am Acad Dermatol.

[15] Lapunzina P, Gairi A, Delicado A, Mori MA, Torres ML, Goma A, Navia M, Pajares IL (2004). Macrocephaly-cutis marmorata telangiectatica congenita: report of six new patients and a review.. Am J Med Genet A.

[16] Martínez-Glez V, Romanelli V, Mori MA, Gracia R, Segovia M, González-Meneses A, López-Gutierrez JC, Gean E, Martorell L, LapunzinaP (2010). Macrocephaly-capillary malformation: Analysis of 13 patients and review of the diagnostic criteria.. Am J Med Genet A.

